# In vitro expression of precore proteins of hepatitis B virus subgenotype A1 is affected by HBcAg, and can affect HBsAg secretion

**DOI:** 10.1038/s41598-021-87529-9

**Published:** 2021-04-14

**Authors:** Aurélie Deroubaix, Anna Kramvis

**Affiliations:** 1 Hepatitis Virus Diversity Research Unit, Department of Internal Medicine, School of Clinical Medicine, Faculty of Health Sciences, University of the Witwatersrand, Johannesburg, South Africa; 2 Life Sciences Imaging Facility, Faculty of Health Sciences, University of the Witwatersrand, Johannesburg, South Africa

**Keywords:** Microbiology, Molecular biology, Diseases, Medical research

## Abstract

HBeAg, a non-particulate protein of hepatitis B virus (HBV), is translated from the precore/core region as a precursor, which is post-translationally modified. Subgenotype A1 of HBV, which is a risk factor for hepatocellular carcinoma (HCC), has unique molecular characteristics in the basic core promoter/precore regions. Carriers of A1 exhibit early HBeAg loss. We sought to further characterize the precore proteins of A1 in vitro. HuH-7 cells were transfected with subgenomic constructs expressing individual precore proteins. Western blot analysis using DAKO anti-core antibody showed the expected sizes and a 1 kDa larger band for P22, P20 and P17. Using confocal microscopy, a cytoplasmic accumulation of HBeAg and precursors was observed with P25-expressing plasmid, whereas P22 localized both in the cytoplasm and nucleus. P20 and P17, which lack the carboxy end of P22 showed strong nuclear accumulation, implicating a nuclear localization signal in the N-terminal 10 amino acids. G1862T, unique to subgenotype A1, is frequently found in HBV from HCC patients. P25 with G1862T showed delayed and reduced HBeAg expression/secretion. Knock-out of core in the replication competent clones led to precore protein accumulation in the cytoplasm/perinuclear region, and decreased HBeAg secretion. Knock-out of precore proteins increased HBsAg secretion but intracellular HBsAg expression was unaffected. Over-expression of precore proteins in *trans* led to decreased HBsAg expression and secretion. Intracellular trafficking of HBV A1 precore proteins was followed. This was unaffected by the CMV promoter and different cell types. In the viral context, precore protein expression was affected by absence of core, and affected HBsAg expression, suggesting an interrelationship between precore proteins, HBcAg and HBsAg. This modulatory role of HBeAg and its precursors may be important in viral persistence and ultimate development of HCC.

## Introduction

Hepatitis B virus (HBV), a member of the family *Hepadnaviridae*, is a small enveloped DNA virus, infecting the liver. Viral persistence leads to various clinical manifestations including liver fibrosis, cirrhosis and hepatocellular carcinoma (HCC). In addition to the structural proteins, which form the capsid (HBcAg) and envelope (HBsAg), and the enzyme, polymerase, all hepadnaviruses encode for the non-particulate protein, HBeAg^1^. Although HBeAg is not required for viral infection, replication or assembly^2–4^, its conservation signifies its important functions^5^. It is required for natural infection^6,7^. In addition to being a biomarker of viral replication, infectivity, inflammation, severity of disease and response to antiviral therapy, HBeAg is a tolerogen and immunomodulator^8,9^. The prevailing hypothesis is that HBeAg has an immunoregulatory/tolerogenic role in serum, whereas when cytosolic, it is a target for the immune system^7,10^. Even at low serum concentrations, HBeAg induces profound tolerance^11^ and it is important in perinatal mother-to-child transmission of HBV, with children born to HBeAg-positive mothers being more frequently and persistently infected^12,13^.

HBeAg is encoded by the precore/core (pre-E) mRNA and like many other secreted proteins, is made from a pre-pro-protein, P25^14,15^. The P25 (25 kDa) has a typical signal peptide, which directs the precursor protein from the cytosol to the secretory pathway^16^. In the endoplasmic reticulum (ER), the amino end is cleaved to yield the P22^17–19^, the pro-protein, which is further processed in the Golgi apparatus by furin cleavage of the carboxyl end, to yield P20. Next, C-terminal cleavage of P20 yields P17, the mature HBeAg, which is secreted or expressed on the surface of the hepatocyte^10,19–22^.

Various mutations within the basic core promoter (BCP) and precore region can affect the expression of HBeAg at the transcriptional, translational and post-translational levels^13,23,24^ leading to differences in clinical manifestation and duration of disease^25^. The tendency of the genome to develop these mutations is dependent on the HBV genotype/subgenotypes of HBV^5,23^.

HBV is endemic in southern Africa with subgenotype A1 prevailing^26^. This subgenotype has a higher hepatocarcinogenic potential compared to (sub)genotypes circulating in the region and HCC develops 6.5 years earlier than in individuals infected with other (sub)genotypes^27^. Compared to other (sub)genotypes, A1 is characterized by the lowest expression of precore/core precursor in the secretory pathway^28^, the lowest HBeAg and core expression and by the lower replicative activity, both in vitro and in vivo^9,29^. Subgenotype A1 has unique molecular characteristics especially within the BCP/precore regions^23,26^ and a very sophisticated way of controlling HBeAg expression^30^, which result in a high rate of HBeAg negativity in carriers of this subgenotype^24,26,31^.

Subgenotype A1 develops mutations that affect HBeAg expression at the transcriptional, translational and post-translational levels^24^. The 1762T1764A mutations, which occur in all (sub)genotypes, affect transcription of the precore mRNA^32^. Positions 1809–1812 from the *Eco*RI site in the Kozak sequence of the preC/C open reading frame (ORF) commonly show mutations in subgenotype A1, which affect HBeAg expression at the translational level^33^. A G to T transversion at position 1862 in the precore region results in a valine to phenylalanine substitution in the –3 position of the signal peptide cleavage site at position 19 of the precursor protein. The phenylalanine, is an aromatic amino acid, which interferes with signal peptide cleavage, a post-translational modification necessary for HBeAg expression^34^. This G1862T mutation occurs almost exclusively in subgenotype A1^23,24^, is more common in HBV from HBeAg-negative than in HBeAg-positive South African carriers^35,36^ and found in HBV isolated from HCC tumor, but not from adjacent non-tumorous liver tissue^35^.

Subgenotype A1 has been studied extensively in our laboratory in order to understand its early HBeAg/anti-HBe seroconversion, its lower replicative and its high hepatocarcinogenic potential. HuH-7 cells transfected with A1 showed a lower expression of the precore/core precursor in the secretory pathway and a higher localization in the nucleus compared to subgenotype A2^28,37^. Cells transfected with A1 showed greater ER stress and an earlier, prolonged activation of the unfolded protein response (UPR) and cells transfected with A1 had increased apoptosis^38^. When G1862T was introduced into a full genome genotype D plasmid, with genotype A precore, driven by a cytomegalovirus (CMV) promoter, it resulted in a 54% reduction in the secretion of HBeAg relative to the wild-type and caused the formation of aggresomes^39^. In the context of a replication-competent subgenotype A1 clone, G1862T diminished HBeAg expression albeit at a lower degree (22%)^38^. The mutant was found to lead to the accumulation of the HBeAg precursor protein in the ER and ERGIC and this accumulation resulted in an earlier activation of the three UPR pathways, leading to increased ER stress without increasing apoptosis^38^.

The objective of the present study was to further characterize subgenotype A1, with and without G1862T, by transfection of HuH-7 cells with subgenomic constructs expressing the precore proteins individually and monitoring their subcellular expression, localization and secretion, to determine whether these:

- Were influenced by overexpression driven by a CMV promoter.
- Were comparable to when cells were transfected with a full genome construct driven by an authentic promoter.
- Were affected when core protein expression was knocked out in the full genome context.
- Affected HBsAg expression and secretion when precore proteins were expressed in *cis* or *trans*.

## Results

### Kinetic analysis of the expression, localization and secretion of HBeAg and its precursors P25, P22 and P20 following transfection of HuH-7 cells with subgenomic constructs

#### An additional band was expressed following expression of P22, P20 and P17 subgenomic constructs

**Figure 1:**
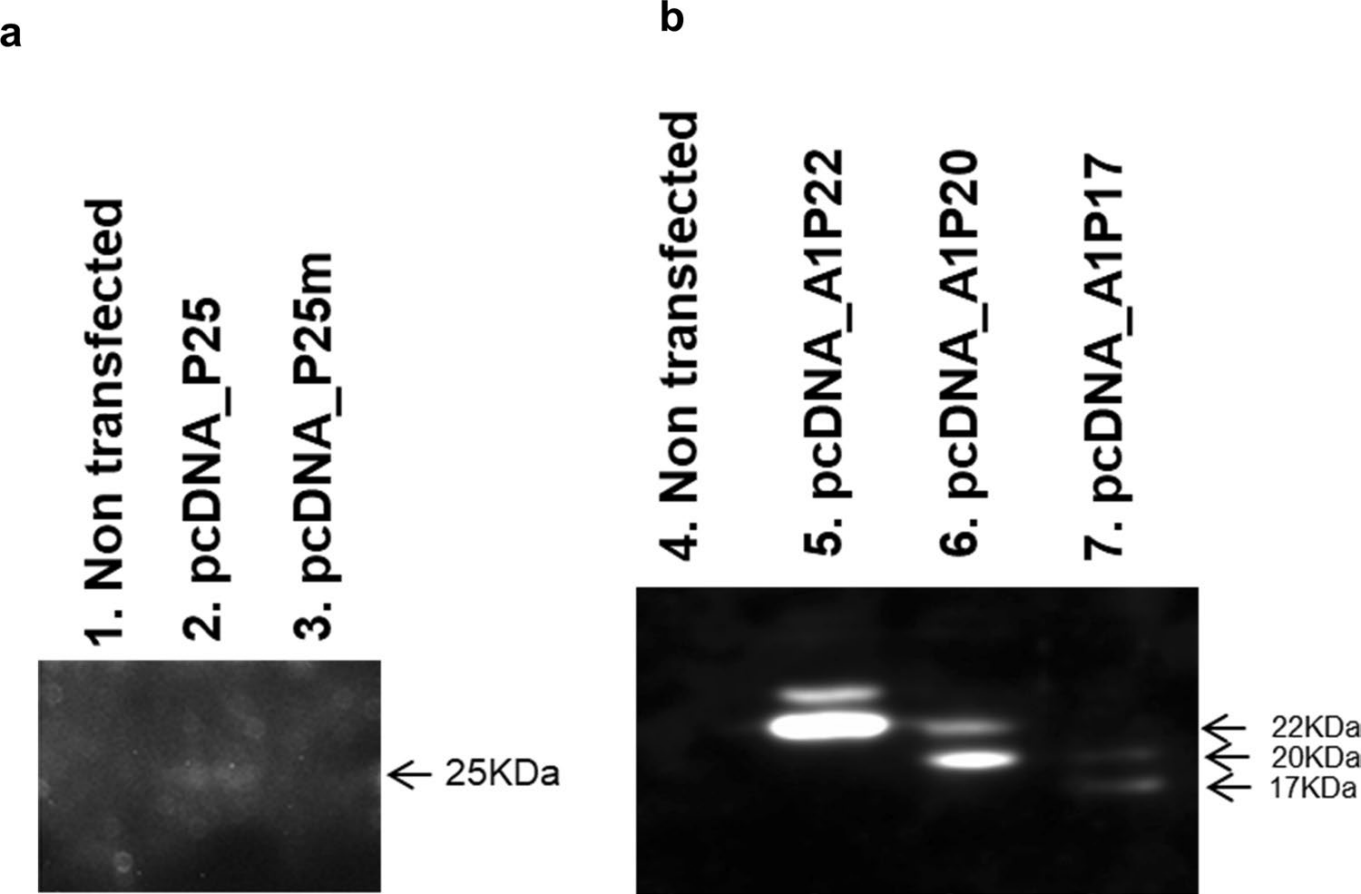
Expression of HBeAg and precursors. Detection of HBeAg and precursors by western blot. HuH-7 cells were transfected with the plasmids indicated at the top of the figure. Cell lysates were analysed on 15% acrylamide gels. Lysates from transfections with [pcDNA_P25, pcDNA_P25m plasmids] (**a**) and [pcDNA_P22, pcDNA_P20, pcDNA_P17 plasmids] (**b**) were loaded in two different gels. Western blots were performed with anti-core antibody and a secondary antibody linked to horse radish peroxidase (HRP). For more clarity, the gels have been cropped. Full-length blots are presented in Supplementary information (Fig. S2).

Subgenotype A1 HBV and each of its precursors (abbreviated, HBeAg/precursors), were expressed individually under the control of the CMV promoter by transfecting HuH-7 cells. (A representation of the post-translational products of P25 and P25m is shown in Fig. S1a and b, respectively). The subgenomic constructs expressed HBV proteins of expected sizes [P25 (25 KDa), P25m (25 KDa), P22 (22 KDa), P20 (20 KDa) and P17 (17 KDa)] (Fig. 1a,b, Fig. S2Aa and S2 Ba). The products of the post-translational modifications of either P25 or P25m were not detected (Fig. 1a), probably due to a lack of sensitivity, or rapid degradation of the proteins. The construct with G1862T expressed at a lower level than the wild-type (Fig. 1b). Expression of P22, P20 and P17 yielded a second band of higher molecular weight (~ P23), (~ P21) and (~ P18), respectively (Fig. 1b).

#### HBeAg and its precursors progressively accumulated in the nucleus over time

**Figure 2:**
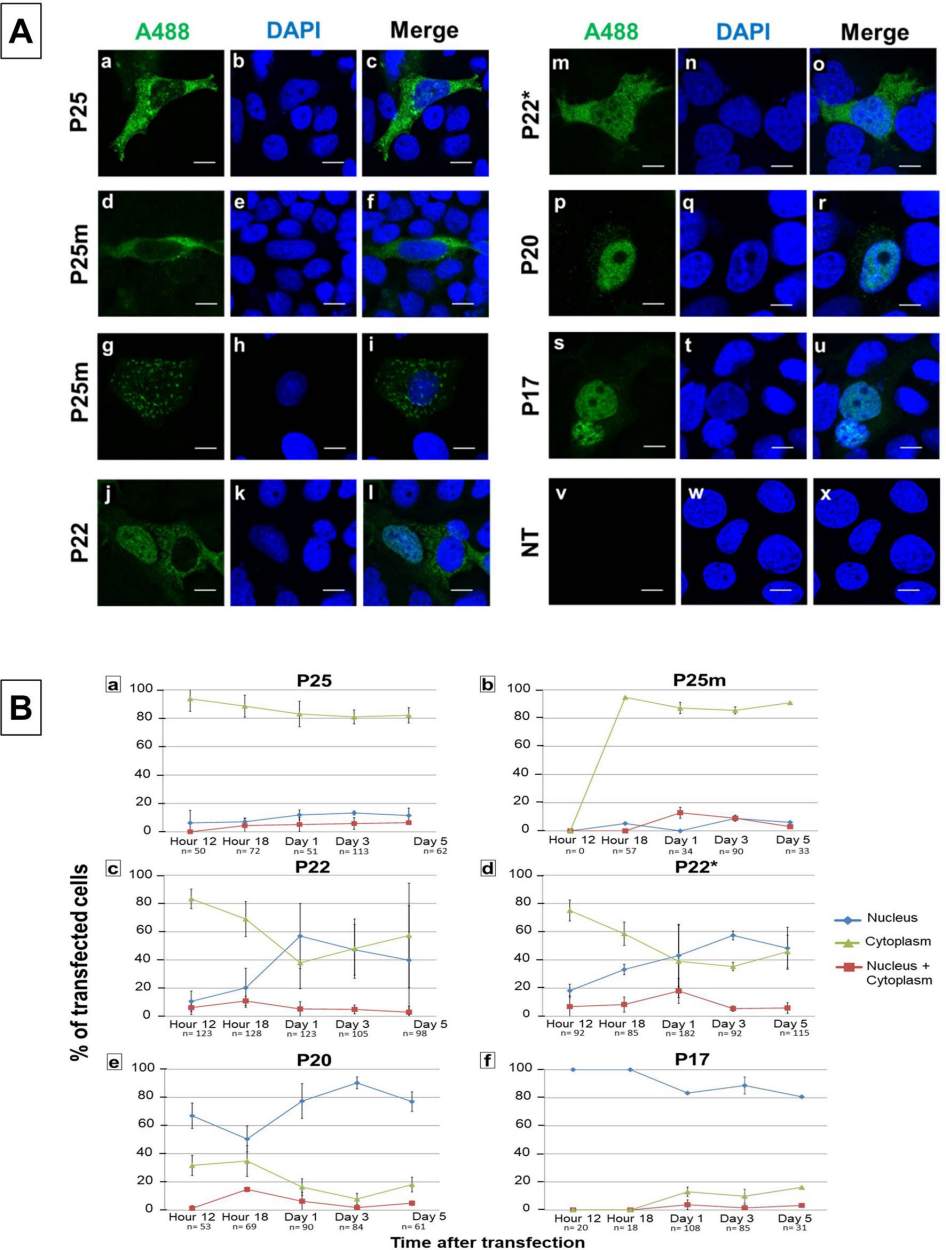
Localization of HBeAg and its precursors over time. (**A**) Intracellular localization of P25, P25m (P25 G1862T), P22, P22*, P20 and P17 proteins from subgenotype A1, after transfection of pcDNA_A1P25 (a–c), pcDNA_A1P25m (d–i), pcDNA_A1P22 (j–l), pcDNA_A1P22* (m–o), pcDNA_A1P20 (p–r), pcDNA_A1P17 (s-u). (*) means the core protein is knocked out. HBV proteins were expressed in HuH-7 cells under the control of the CMV promoter. Cells were immunostained with a polyclonal rabbit anti-HBc antibody (DAKO, (a,d,g,j,m,p,s,v)) and viewed with a confocal microscope at day 3 post-transfection. Nuclei were visualized by DAPI staining (b,e,h,k,n,q,t,w); merge (c,f,i,l,o,r,u,x). *NT* non-transfected cells. (**B**) Determination of concentration of HBeAg and precursors depicted as quantitative results of 2 to 4 experiments at 12 h, 18 h, day 1, day 3 and day 5 post-transfection. The lines show the predominant localization of HBeAg and its precursors. Accumulation in nucleus or cytoplasm shown in blue or green, respectively. Red lines show an equal distribution between the nucleus and the cytoplasm. The numbers (n) below each graph indicate the number of transfected cells counted.

Localization of HBeAg and precursors was then followed by immunostaining and confocal microscopy during a kinetic (Fig. 2A). It should be noted that the antibodies used in this study do not differentiate between core and precore proteins.

In our in vitro experiments with subgenomic constructs, core protein was expressed in frame with precore. Moreover, as precore is known to interact with core protein and form heterocapsids^40^, core protein could thus have an effect on precore localization. To prevent the expression of core protein by each of the plasmids expressing HBeAg/precursors the core start codon was mutated, by site-directed mutagenesis. No difference in the localization of proteins was observed between constructs either expressing or not expressing HBcAg. Figure 2Am and Fig. 2Bd show the results for P22*, compared to Fig. 2Aj and Fig. 2Bc for P22.

**Figure 3:**
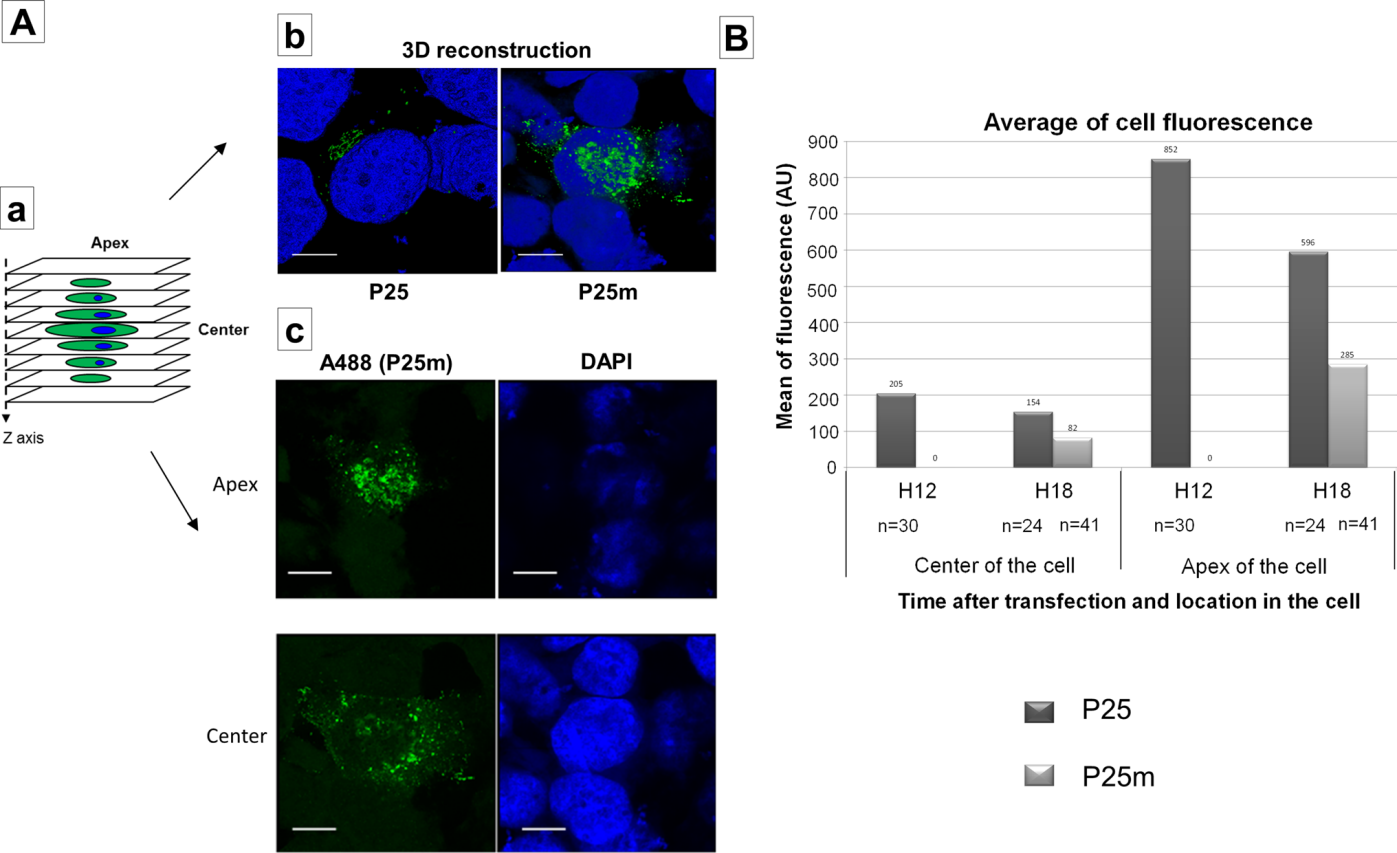
3D representation and comparison of fluorescence intensity after expression of P25 vs P25m. (**A**) Cells from Fig. 1 were used to realize z-stacks by confocal microscopy. 30 cells (hour 12), 24 cells (hour 18 for P25) and 41 cells (hour 18 for P25m) were taken. (a) Z-stack representation. (b) Z-stacks of cells were taken with Airyscan and 3D reconstructions was performed with Zen software. It shows the localization of P25 and P25m at different angles, laterally for P25 and from the apex for P25m. Scale bars = 10 µm. (c) An example of picture at the center and the apex for one cell transfected with P25m at 18 h after transfection. (**B**) The mean of fluorescence were measured for each cell in the plane at the center of the cell and at the apex of the cell. Dark grey, P25; light grey, P25m. n = number of transfected cells.

A kinetic was performed early (6, 12 and 18 h) and on days 1, 3 and 5, after transfection. The fluorescence observed by confocal microscopy in transfected cells (Fig. 2A) was quantified in both the nucleus and cytoplasm, and cells were classified depending on the ratio of the mean of fluorescence between these two cellular compartments (R = N/C, with R > 1, accumulation of fluorescence in the nucleus, R < 1 cytoplasmic accumulation or R = 1, equal distribution between the two compartments, Fig. 2B).

For all transfections, protein expression was first observed at 12 h after transfection. Over a period of 5 days, the majority of the cells had a diffuse cytoplasmic localization of P25 (and post-translational products) with an accumulation near the nucleus, possibly in the ER compartment (Fig. 2Aa). Approximately 94% of the cells had cytoplasmic accumulation of P25 at 12 h. After this, there was a slight decrease in cytoplasmic localization to 80% and a slight increase of the nuclear accumulation of P25 (10%) at days 1, 3 and 5 post-transfection (Fig. 2Ba).

Means of R values are indicated in Fig. S3a (t-test, p-value = 0.0280369 between 12 h and day 5, n = 3 experiments-results were statistically different between the first and last time point of the kinetic), showing nuclear import of P25 or the products of its post-translational modifications. From this experiment it was not possible to differentiate between P25, P22, P20 or P17. If what is being detected are the products of post-translational modification, these are only a minor proportion because as opposed to what was observed for P22, P20 and P17, the N/C ratio for P25 is always < 1.

P22 showed a strong accumulation in the cytoplasm early after transfection (Fig. 2Bc, Fig. S3a). At 18 h, although it remained mostly in the cytoplasm, there was a noticeable decreased localization in this compartment, together with an increase in nuclear localization (69% C, 11% N/C, 20% N). About half the cell population had an accumulation of P22 in the nucleus and the other half had an accumulation in the cytoplasm over 5 days. However, the standard deviation was very high for these 3 days, showing a high variation of localization between the experiments, with either a strong accumulation in the nucleus, or a strong accumulation in the cytoplasm, or an equivalent distribution between the 2 compartments, as illustrated in Fig. 2Aj-l. P22* showed the same distribution than P22 in the cells over 5 days, except that the standard deviation was not as high as for P22 (Fig. 2Bd and S3b). Moreover, we observed a delay in the pattern of expression when comparing P22 and P22*. This observation may suggest that core protein was also expressed and was transported to the nucleus in the early stages, while HBeAg was imported to the nucleus later. The high variability may be due to core protein expression, as it is known to shuttle constantly between the nucleus and the cytoplasm. However, it would not be expected to be highly expressed considering that in our constructs, core protein and precore/core precursor are expressed from the same mRNA, and the ribosomes would preferentially use the first start codon. N/C ratios confirmed a progressive accumulation of P22 and P22* in the nucleus (Fig. S3b) (t-test, p = 0.00005 and p = 2.27 e-9 for P22 (n = 3 experiments) and P22* (n = 4 experiments), respectively) when comparing the N/C ratios between hour 12 and day 5 post transfection, showing statistically different results (Fig. S3b)).

P20 and P17 proteins showed a strong (sometimes exclusive), progressive nuclear accumulation over 5 days (Fig. 2A, p and s respectively, Fig. 2Be and Fig. S3c). P20 accumulated in the nucleus in 67% of cells at 12 h, then decreased to 50% at 18 h (Fig. 2Be). Thereafter, the protein accumulated in the nucleus by 3 days and decreased again at day 5 (Fig. 2Be). P17 showed a stronger nuclear accumulation (100% N 12 h and 18 h) with a slight decrease but still with a strong nuclear accumulation on days 1, 3 and 5 after transfection. (R > 1 for P20 and P17 (Fig. S3c)).

In order to preclude the possibility that the CMV promoter affected the localization of HBeAg/precursors, HuH-7 cells were also transfected with constructs driven by the HBV authentic BCP/precore promoter (pcDNA_BCP_p25). As shown in Fig. S4, the promoters did not affect the expression of the proteins.

To provide further evidence that the CMV promoter does not affect protein localization, 1,4-galactosyltransferase (Golgi7), a protein specific to the Golgi, was over-expressed using a CMV promoter. This protein is only expressed in the Golgi compartment of the secretory pathway^41^. After compensating for background fluorescence, Golgi7 was as expected found exclusively in the secretory pathway and not in the cytoplasm or the nucleus (Supplementary Fig. S5).

In order to assess if a tag could affect the localization of HBeAg/precursors, we cloned an HA-tag onto the P22 C-terminus, designated as P22-HA or to the N-terminus, designated as HA-P22; and compared the localization over 5 days post-transfection. Addition of the HA-tag to the carboxyl end (Fig. S6a–c) did not change the localization when compared to P22 lacking tag (Fig. 2Aj-l); and gave a similar distribution of fluorescence between the nucleus and cytoplasm. Localization was also observed in the plasma membrane (Fig. S6d-f). However, when the HA-tag was included on the amino end, no nuclear localization was observed in all the microscopic fields examined (Fig. S6g-i). Similar results were obtained when the HA-tags were inserted on the amino and carboxyl ends of P25, P20 and P17 (see ‘Availability of materials and data’ for more information).

In order to confirm that the cells used for transfection did not influence the localization of the proteins, HepG2 cells were transfected with P22. As observed when HuH-7 cells were transfected, at 12 h post-transfection, the protein accumulated mostly in the cytoplasm of HepG2 cells (76%) (Supplementary Fig. S7).

#### G1862T did not induce a change in P25 localiation but delayed P25 expression, leading to decreased P25 accumulation in the secretory pathway with reduced HBeAg expression

The introduction of the G1862T mutation did not affect the localization of p25 (Fig. 2Aa vs 2Ad), although an increased number of cells, compared to the wild-type, showed granulated staining dispersed through the cytoplasm (Fig. 2Ag). This may suggest an interaction with subcellular organelles or aggregates in the cytoplasm. However, the introduction of the G1862T did result in a delay in the expression of the mutant protein relative to the wild-type p25. At 12 h post transfection, no cells expressed mutated P25 (Fig. 2Bb), whereas some cells expressed wild-type P25 (Fig. 2Ba). The earliest expression of P25m was observed 18 h after transfection. Subsequently, the expression of P25m followed the same pattern as P25, with cytoplasmic localization in about 90% of cells at 18 h, and at days 1, 3 and 5 (Fig. 2Bb).

N/C ratios for P25 and P25m followed the same pattern (Fig. S3a) (t-test: p = 0.04, between 18 h and day 5, signifying a statistically significant difference between the first and last time point of the kinetic). The N/C results were not statistically different between P25 and P25m (p = 0.593675; n = 3 experiments), demonstrating that the G1862T mutation does not affect the trafficking of P25 between the nucleus and the cytoplasm. In general, when the cells were transfected with the G1862T construct, the intensity of fluorescence in the cells was decreased by a factor of 2, relative to the wild-type (see 'Availability of materials and data' for more information).

Z-stacks (Fig. 3Aa) and 3D reconstructions were performed. The similar distribution of P25 and P25m was visualized in 3D (Fig. 3Ab). The mean of fluorescence was measured in 2 planes at 12 and 18 h after transfection: particularly at the middle and at the apex of the cell, where an accumulation of fluorescence was observed, in a structure resembling the secretory pathway (Fig. 3Ac). P25m expressing cells have a lower mean of fluorescence, but the relative distribution of the proteins between the centre and the apex of the cells was comparable. As seen in Fig. 2, P25m expression occurred later, at 18 h after transfection in both planes (Fig. 3B).

**Figure 4:**
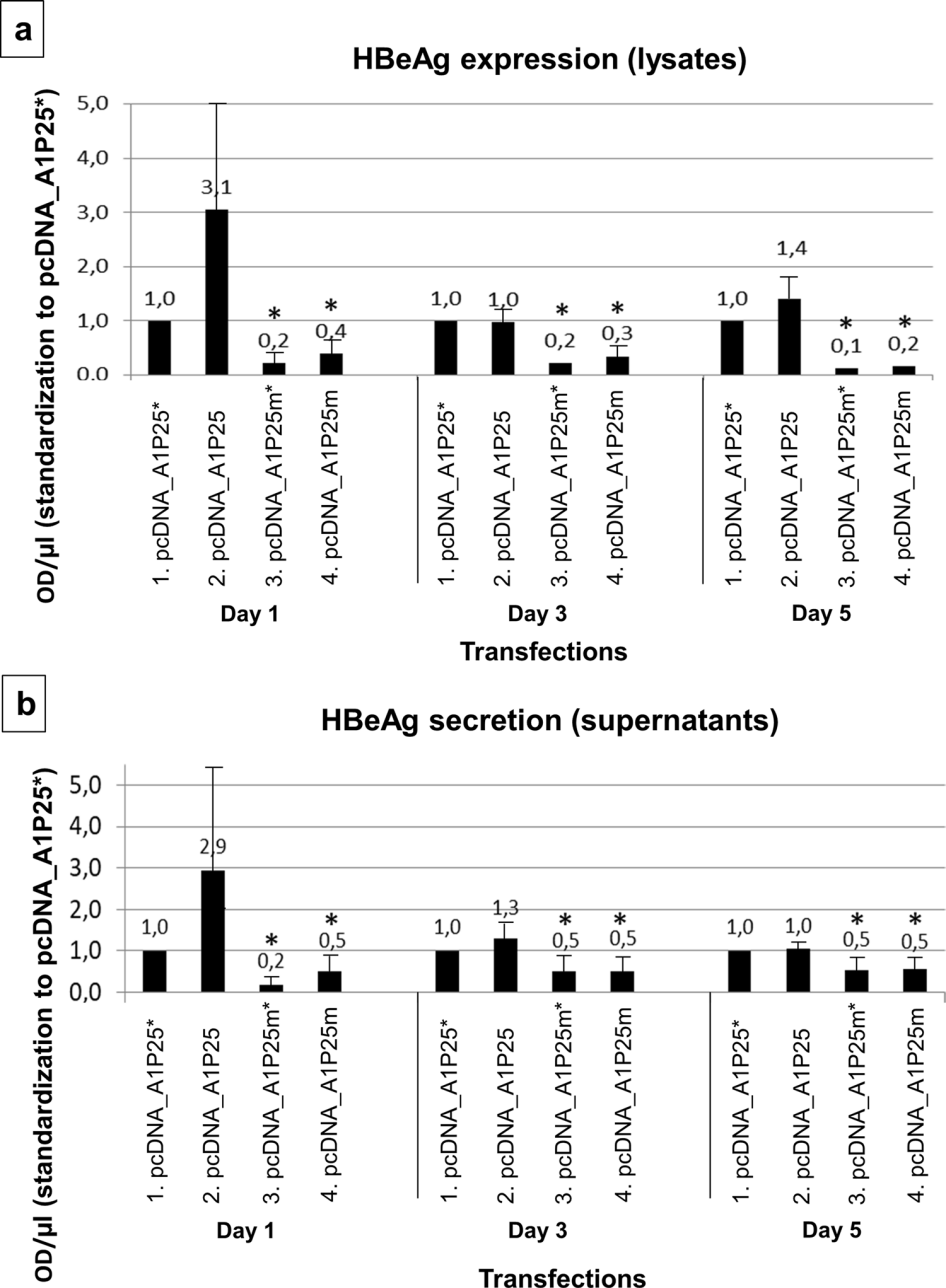
HBeAg expression and secretion—ELISA quantifications. After transfection of the plasmids expressing P25, P25* P25m and P25m*, cell lysates (**a**) and supernatants (**b**) were collected day 1, day 3 and day 5 post-transfection and subjected to an ELISA test for HBeAg. Results were standardized to P25*. Results of 3 experiments. (*) above columns means results for P25m* and P25m are significatively different from P25* and P25, respectively. Results are not significatively different between P25* and P25, and between P25m* and P25m.

The ELISA for HBeAg confirmed that on all days cellular expression of HBeAg was higher for P25 compared to P25m (Fig. 4a), with the knock-out of HBcAg expression not affecting either the amount of expression or the ratio of P25/P25m expression. When comparing P25*/P25m* to P25/P25m the trends were almost identical (Fig. 4a).

HBeAg was detected in the supernatant of medium collected from cultured cells (Fig. 4b), only after transfection with pcDNA_A1P25, but not following transfection with subgenomic plasmids expressing P22, P20 and P17 (see ‘Availability of materials and data’ for more information). As P25 has the signal peptide on its amino end, which targets it to the ER, it is processed to HBeAg, which is then secreted into the supernatant. In the presence of G1862T, HBeAg secretion was impaired by a factor 5.8 on day 1, a factor 2.6 on day 3 and a factor 2 on day 5 compared to the wild-type (Fig. 4b lanes 4 vs 2). The introduction of the mutation suppressing core expression did not change the secretion pattern (Fig. 4b, lanes 3 vs 1).

We also included an additional control, where we transfected a HBV core protein expressing plasmid (pcep21) and performed the anti-HBeAg ELISA assay 3 days post-transfection. In the 3 experiments conducted, no positive results were obtained, confirming that, even if core protein is secreted, it is not detected by the anti-HBeAg ELISA kit (see 'Availability of materials and data' for more information).

### HBV core protein impairs HBeAg and precursors’ expression, localization and secretion in the viral context

**Figure 5:**
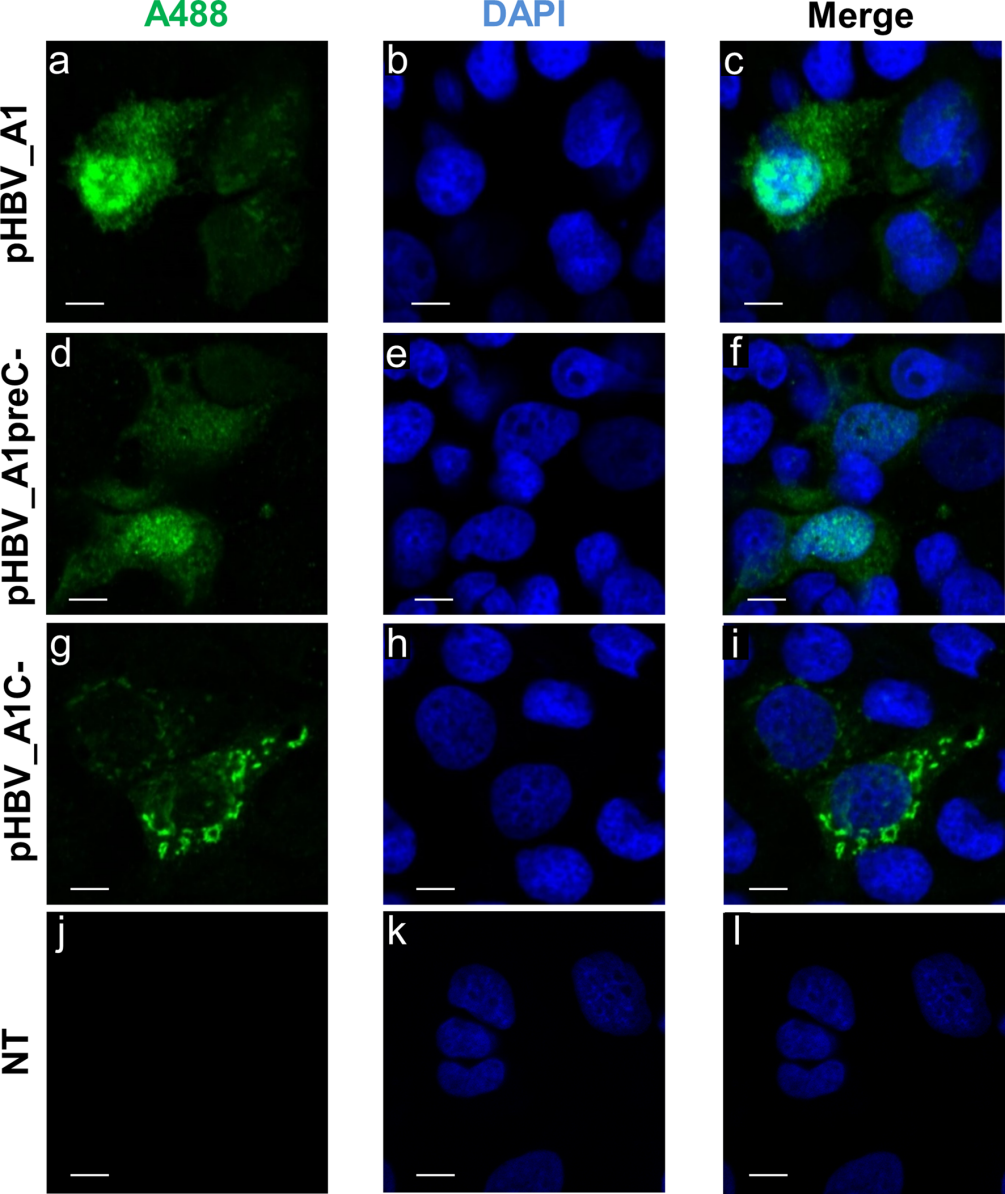
Intracellular localization of HBeAg and its precursors following transfection with a subgenotype A1 replication competent clone. Cells were transfected with pHBV_A1, pHBV_A1preC- (HBV A1 replication competent clone where ATG of precore has been mutated) and pHBV_A1C- (HBV A1 replication competent clone where ATG of core has been mutated). Cells were immunostained with a polyclonal rabbit anti-HBc antibody (DAKO, (**a,d,g,j**) and viewed with a confocal microscope on day 3 post-transfection. NT: non-transfected cells (**j,k,l**). Nuclei were visualized by DAPI staining (**b,e,h,k**); (**c,f,i,l**): merge. Bars, 10 µm.

We followed the expression of HBeAg and its precursors by comparing transfection with complete genome constructs and the subgenomic constructs. When cells were transfected with pHBV_A1, heterogenous staining was observed (Fig. 5a–c): cells with accumulation of fluorescence in the cytoplasm, cells with equal fluorescence in the nucleus and cytoplasm and cells with accumulation in the nucleus. This staining was diffuse and finely granular. Some cells showed small aggregates around the nucleus, which may represent expression in the secretory pathway. The localization of preC/C/HBeAg was the same as that observed previously, when these proteins were expressed with the CMV promoter (Fig. 3). More nuclear accumulation was due to the presence of core protein, which has a nuclear localization signal; and also, the antibodies used cross-react with HBcAg and HBeAg.

When we transfected with pHBV_A1preC- (Fig. 5d–f), which only expresses core protein due to mutation of precore ATG, while we observed the same heterogeneity in localization, no aggregates were evident in the perinuclear region (Fig. 5d–f). The core protein localized in the entire cell, with alternate accumulation in the nucleus or cytoplasm. This is in accordance with the literature, where core protein is known to shuttle between the nucleus and the cytoplasm, due to the presence of NLS and Nuclear Export Signal. In contrast, when transfected with pHBV_A1C-, large aggregates were observed in the perinuclear region (Fig. 5g–i). Although some fluorescence was observed in the nucleus, this was not prominent. The pattern of expression was the same as when cells were transfected with subgenomic pcDNA_A1P25*. In the absence of core protein, the intensity of fluorescence was 2.6 fold higher ((Fig. 5g–i) versus (Fig. 5a–c)), intimating that core protein may influence the expression of HBeAg and precursors.

**Figure 6:**
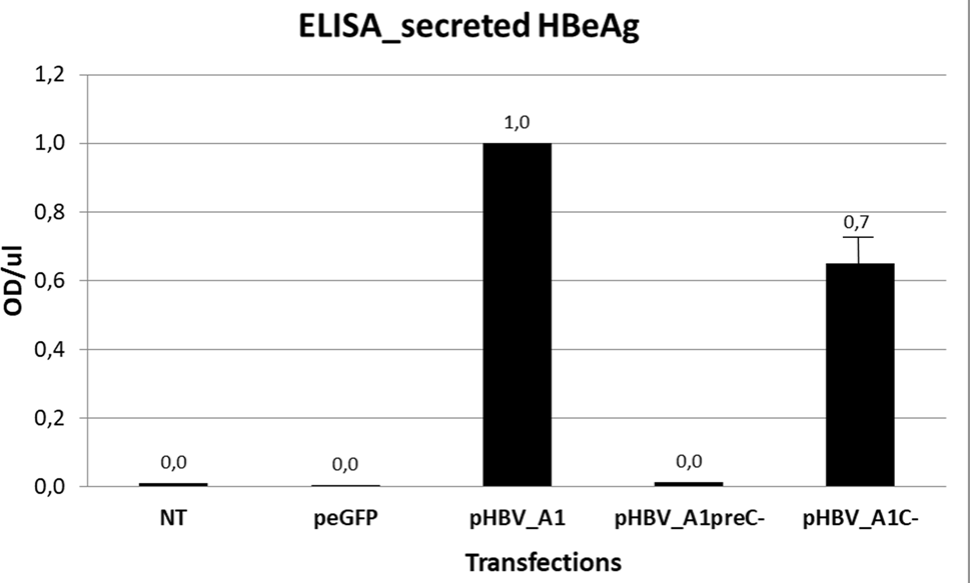
HBeAg secretion in viral context. pHBV_A1, pHBV_A1preC- and pHBV_A1C-were transfected in HuH-7 cells. 3 days post-transfection, supernatants of cells were collected and subjected  to anti-HBeAg ELISA. Results were normalized to pHBV_A1. Average of 3 experiments.

We compared the secretion of HBeAg in the supernatants transfected with pHBV_A1, pHBV_A1preC- and pHBV_A1C- (Fig. 6), where results were normalized to the secretion of HBeAg with pHBV_A1. Transfection of cells with pHBV_A1preC- abolished HBeAg secretion. Transfection of cells with pHBV_A1C- decreased HBeAg secretion by 30% (results statistically different, t-test, p = 0.0065, n = 3 experiments). Thus absence of core protein expression lowered HBeAg secretion. As the ELISA kit did not detect core protein in the supernatant, this cannot be attributed to loss of core protein expression. Further, the ELISA measurements are in agreement with the confocal microscopy findings, where knock-out of core protein led to an accumulation of HBeAg and precursors in the perinuclear region (Fig. 5). The core protein, which interacts with HBsAg and forms new virions^42–45^, may possibly interact with HBeAg and its precursors, facilitating the secretion of HBeAg.

### HBeAg and its precursors impair HBsAg expression and secretion in the viral context

**Figure 7:**
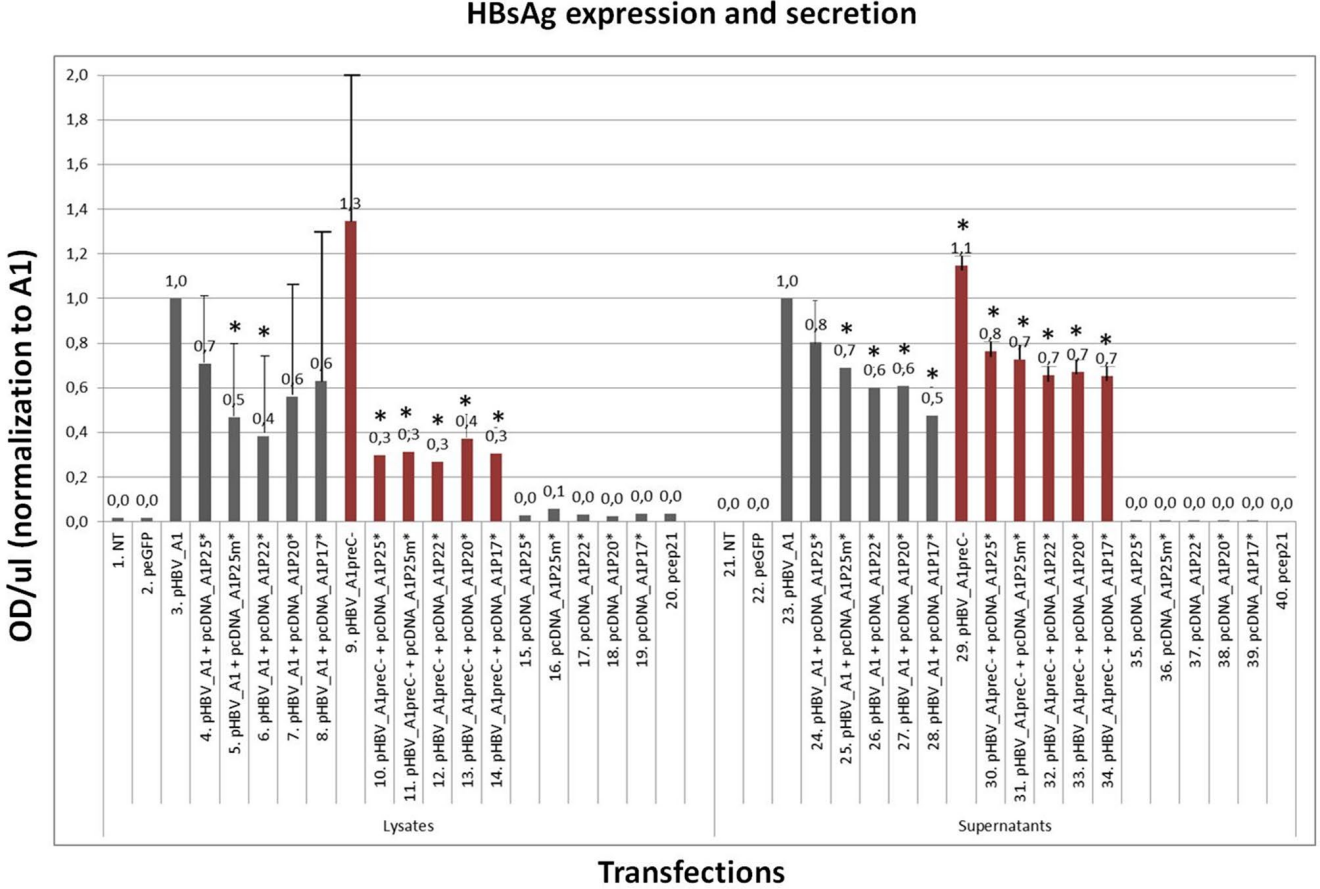
Effect of HBeAg and precursors on HBsAg expression and secretion. (**A**) Co-transfections of the subgenomic plasmids with pHBV_A1. HBV A1 replication competent clone (pHBV_A1) and A1 clone with knock-out of preC (pHBV_A1preC-) were transfected in HuH-7 cells. Cells were also co-transfected with pHBV_A1 and each of the plasmids pcDNA_A1P25*, pcDNA_A1P25m*, pcDNA_A1P22*, pcDNA_A1P20* or pcDNA_A1P17*. 3 days post-transfection, lysates and supernatants of cells were collected and subjected to anti-HBsAg ELISA. Results were normalized to pHBV_A1 for supernatants and for lysates. (**B**) Co-transfections of the subgenomic plasmids with pHBV_A1preC-. HuH-7 cells were co-transfected with pHBV_A1preC- and each of the plasmids pcDNA_A1P25*, pcDNA_A1P25m*, pcDNA_A1P22*, pcDNA_A1P20* or pcDNA_A1P17*. 3 days post-transfection, lysates and supernatants of cells were collected and subject to anti-HBsAg ELISA. Results were normalized to pHBV_A1preC- for supernatants and for lysates. (*) above each column indicates significant differences with pHBV_A1. Result of average of 4 independent experiments. *NT* non-transfected cells.

The effect of HBeAg and its precursors on HBsAg expression was investigated. Results were normalized to the expression/secretion of HBsAg by pHBV_A1 (Fig. 7). When expression of precore was knocked out (pHBV_A1preC-) there was an increase in HBsAg expression and secretion, relative to the wild-type (pHBV_A1) (Fig. 7, lanes 9 and 29). Next we over-expressed HBeAg and precursors by co-transfection of either pHBV_A1 or pHBV_A1preC-with each of the CMV plasmids, pcDNA_A1P25*, pcDNA_A1P25m*, pcDNA_A1P22*, pcDNA_A1P20*, pcDNA_A1P17*. Co-transfection of pHBV_A1 with pcDNA_A1P25* did not affect HBsAg expression and secretion significantly (Fig. 7, lane 4 and 24); whereas co-transfection of pcDNA_A1P25m* did (Fig. 7, lane 5 and 25). Moreover when pcDNA-A1P25* was co-transfected with pHBV_A1preC- there was a significant decrease in HBsAg expression and secretion (Fig. 7, lane 10 and 30), with the reduction being significantly greater in the lysates compared to supernatants (70% and 20% decrease, respectively). When pcDNA_A1P22* was co-transfected with pHBV_A1, there was a decreased of HBsAg expression by 60% (Fig. 7, lane 6) but co-transfection with pcDNA_A1P20* and pcDNA_A1P17* did not have any effect (Fig. 7, lane 7 and 8). On the other hand, over-expression of either P22*, P20* or P17* with pHBV_A1preC- decreased HBsAg secretion by 30% (Fig. 7, lanes 32, 33 and 34). In the presence of G1862T mutant, HBsAg expression decreased a further 20% relative to when cells were co-transfected with pcDNA_A1P25* (Fig. 7, lane 5) and HBsAg secretion decreased by a further 10% (Fig. 7, lane 25). Thus over expression of HBeAg and its precursors decreased HBsAg expression and secretion. When the subgenomic plasmids expressing HBeAg and its precursors were co-transfected with pHBV_A1preC-, the reduction of HBsAg expression was enhanced (Fig. 7 lane 10) and even p20 and p17 had a significant effect (Fig. 7, lanes 13 and 14). Therefore, the effect of HBeAg and its precursors on the expression of HBsAg, was enhanced when they were co-transfected with HBeAg-negative replication competent clone and the HBeAg was expressed only in* trans* rather than in *cis*.

## Discussion

Almost 50 years since its discovery^1^, HBeAg and its properties continue to fascinate. Even though its role as an immunomodulator and tolerogen is being elucidated and understood^8,9^ many questions remain unanswered. HBeAg is a protein, which is expressed by post-translational modification of a preproprotein, with a number of intermediary precursors^17,19,20,22^, which have survived its long evolutionary history^46^. The aim of present study was to express the precursors of HBeAg of subgenotype A1 HBV, individually in vitro, in order to follow their expression and to further understand their roles. Even though in vitro expression systems have their limitations they provide us with models that allow us to follow HBV infection and to dissect out complex biological processes^47^. They allow us to determine the roles of the individual HBeAg proteins, without the influence of other HBV proteins, including the capsid HBcAg and the envelope HBsAg proteins and also to determine their effect on the latter proteins and vice versa.

As shown in Fig. 1 the size of the HBeAg (P17) and its precursors (P25, P22, P20) expressed from the subgenomic constructs of subgenotype A1, agreed with those predicted by their coding sequences and with the findings of others^48,49^. The introduction of the G1862T mutation in the subgenomic P25 led to decreased HBeAg expression as previously observed in replication competent clones belonging to subgenotype A1^38^ but not subgenotype A2 clones^50^. In agreement with previous studies^48,49,51^, two species of P22 (P22 and P23), P20 (P20 and P21) and P17 (P17 and P18) were observed (Fig. 1b). These have been speculated to be the result of post-translational covalent modification occurring in the cytoplasm^49^. P23 is confined to the cytoplasmic fraction whereas 20–30% of P22 was found in the microsomal fraction, with the remaining portion found in the cytoplasm^49^. It has been suggested that the difference between the two species is as a result of a modification on carboxy terminus, within the arginine-rich charged domain, catalyzed outside the microsomal fraction, by an enzyme present in both plants and animals^49^.

Although P25 has an affinity for the ER^51^, its aborted translocation results in its presence in the cytosol^16,49^. Similarly, following signal peptide cleavage of P25, 70–80% of P22 can translocate to the cytoplasm because of uncoupling from subsequent translocation events^49^. Post-translational modification of P22 occurs in the post-ER Golgi compartment to give rise to P20 and P17^52^, that are confined to the ER and lead to the secretion of HBeAg (Fig. S1). However, by expressing P20 and P17 from subgenomic fragments we sought to follow their fate from the cytoplasm as these proteins are effectively P22 with the deletion of the carboxyl end. No HBeAg was expressed in the supernatant when Huh-7 cells were transfected with subgenomic plasmids pcDNA_A1P22, pcDNA_A1P20 and pcDNA_A1P17 (see 'Availability of materials and data' for more information), confirming that HBeAg expression occurs via the ER pathway and not via an alternative pathway such as the endosomal sorting complex required for transport (ESCRT)/multivesicular bodies (MVB) as seen for HBV virions^53,54^ and filaments^55^ or the ESCRT-independent manner regulated by Alix used by HBcAg containing naked capsids^56^.

Localization was comparable between experiments conducted with plasmid constructs with either an authentic or CMV promoter. Expression of P22 was similar in HuH-7 and HepG2 cells. Thus localization was independent of either the promoters or the cell culture system. Because core is expressed in-frame and the antibodies used do not differentiated between core and precore proteins, we also followed the expression of HBeAg/precursors using plasmids with knock-out of the core ATG (P25*, P25m*, P22*, P20* and P17*). No difference in the localization of proteins was observed between constructs expressing and not expressing HBcAg. Thus, co-expression of core protein did not influence the localization of HBeAg/precursors or interfere with the immunostaining. HA tags, however, did influence the localization of HBeAg and its precursors. When the HA tag was inserted on the amino end there was no translocation of P22 into the nucleus and when it was on the carboxyl end there was translocation into the nucleus (Fig. S6).

In the early hours post-transfection, P25 was expressed in the cytoplasm only, with increase in nuclear/cytoplasmic ratio (R) over time (Fig. 2Aa, Ba, S3a). On the other hand, P22 demonstrated a stronger accumulation in the nucleus with R reaching 1.4 on day 5. The signal peptide on the N-terminal of P25 targets it to the ER, and it has been found in the nucleus and the cytoplasm of *Xenopus* oocytes after micro-injection of HBV mRNA^48^. When the translocation and expression of P25 is aborted, P25 is released into the cytoplasm^49^, where it equilibrates passively between the cytoplasm and the nucleus^48^. Similarly a portion of P22 can escape the secretory pathway in COS and CV-1 cells^16^ via endoplasmic-reticulum-associated-degradation (ERAD) in HuH-7 cells and enter the cytoplasm and the nucleus^57^. P25 and P22 differ in size because P22 lacks the signal peptide found on P25 and they share the nuclear localization sites (NLS) on their carboxyl ends^58,59^, which are also present in core protein^58^. Partially purified HBeAg has been shown to interact with DNA^60^. The frequency of nuclear localization of HBeAg precursors was higher in HuH-7 cells transfected with subgenotype A1 replication competent clones compared to A2 or D3^28^. The carboxyl-terminal arginine rich domain can bind nucleic acids as shown by P25 expressed in *Escherichia coli*^61^, and to interact with the nuclear transport factor, karyopherin α1^62^. Liver biopsies from patients with chronic hepatitis show a higher frequency of preC/C/HBeAg in the nucleus compared to patients with acute hepatitis^63,64^ and thus nuclear accumulation may lead to viral persistence^57^. P25 and P22 in the nucleus may have roles that require further investigation.

It has been proposed that the 10 mer or region 2^5^, which remains following signal peptide cleavage is important for nuclear transport of P22^16^ and may act alone or in combination with the carboxyl end NLS^65^. It has been proposed that the 10 mer alters the tertiary structure causing the exposure and activation of the NLS on the precore^59^. In order to address this, we expressed P20 and P17, which represent the products of post-translational modification of P22, where the carboxyl end is cleaved by furin^19,20,22^. P20 and P17 proteins showed a strong and sometimes exclusive nuclear accumulation over time showing that these proteins can translocate to the nucleus in the absence of the carboxyl end (Fig. 2A). This, however, contrasts with experiments using precore-expressing plasmids tagged with HA on the amino end, which showed no nuclear localization, in absence of the carboxyl end (P17)^62^. This may be as a result of differing experimental methods. As we have shown for P22 (Fig. S6) and P25, P20 and P17, when the HA was tagged on the carboxyl end there was translocation to the nucleus but not when the HA-tag was on the amino end (Fig. S6). Thus the HA-tag on the amino end may prevent the translocation of the precore proteins to the nucleus as shown by others, even in the presence of the carboxyl end^66^. It should be noted that P20 and P17 are not expected to localize in the cytoplasm because retro-transport from the Golgi/ER following post-translational modification of P22 has not been shown to occur^52^. Nevertheless, one study showed that HBeAg (P17) co-localizes with Toll/Il-1 receptor (TIR)-containing proteins in the cytosol^67^. Thus our findings of nuclear localization of P20 and P17 should be interpreted taking these caveats into account.

The marked reduction of expression and secretion of HBeAg in the presence of G1862T, with or without HBcAg expression by the subgenomic constructs, agrees with studies using G1862T mutant replication competent clones transfected in HuH-7 cells^38^. In contrast, when core protein expression was knocked out in the replication competent clones (pcDNA_A1C-), HBeAg accumulated in the perinuclear region (Fig. 5) and secretion was significantly reduced (Fig. 6). Moreover, in the absence of core, movement of P22 to the nucleus occurred later (Fig. 2B). Evidently P22 movement into the nucleus can occur independently of core protein as previously demonstrated using high resolution laser scanning confocal microscopy and Airyscan^68^. The difference in the localization between pcDNA_A1P25 and pcDNA_A1P25* was not as pronounced as between pcDNA_A1 and pcDNA_A1C-. These differences would be expected because in the transfection with the subgenomic constructs, HBeAg and HBcAg are translated from the same mRNA (with the second ATG initiating translation less efficiently). On the other hand, in the viral context, translation of HBeAg and HBcAg occurs from the precore mRNA and pregenomic RNA, respectively. Although knocking out core expression would result in the absence of infection, these experiments allowed us to demonstrate that P22 moves into the nucleus independently of core protein, at a later stage during the course of infection and that in the absence of core, HBeAg expression was reduced. A plausible explanation for this reduction in HBeAg expression is that in the absence of core, precore proteins in the cytosol form dimers instead of heterocapsids with core^40,66,69^. Retention of the precore proteins in the cytosol would diminish the passage of the precursors through the ER, which is necessary for HBeAg secretion.

When expression of precore was knocked out (pHBV_A1preC-) there was an increase in HBsAg expression and secretion relative to the wild-type (pHBV_A1) (Fig. 7). Moreover, when we co-transfected a replication competent clone with subgenomic plasmids expressing HBeAg or its precursors there was a negative effect on HBsAg expression (Fig. 7). This negative effect was enhanced when they were co-transfected with HBeAg-negative replication competent clone and the HBeAg was expressed only in* trans* and not in* cis* (Fig. 7). This decrease in HBsAg, which is a measure of viral replication, correlates with the findings of others who showed that in the absence of a functional precore gene there was an increase in viral replication^40,70^. Moreover, reduced expression of precore because of reduction in transcription of the precore mRNA also resulted in enhanced viral replication^32^ and precore protein was found to inhibit viral replication in transgenic mice^71^. Scaglioni and colleagues also showed a marked reduction in viral replication when either P18 (~ P17) and P22 were co-expressed with wild-type HBV i.e. in* trans*^40^. More recently reduced viral secretion was observed when precore assembled with core to form heterocapsids^66^.

When the subgenomic constructs were co-transfected with pHBV_A1preC-, the HBeAg and its precursors were expressed in *trans* and only P25 enters the ER. P22, P20 and P17 remain in the cytoplasm and are not post-translationally modified because they lack the signal peptide directing P25 to the ER. When the subgenomic constructs were co-transfected with pHBV_A1, HBeAg and its precursors were expressed both in *cis* and in *trans*. As conjectured for HBeAg, the formation of heterocapsids could also be a conceivable explanation for the reduced HBsAg expression observed when subgenomic clones expressing HBeAg and its precursors were co-transfected with replication competent clones pHBV_A1 and pHBV_A1preC-. P22 and P18 (~ P17) have been shown to form unstable heterocapsids with core protein, lacking nucleic acid and thus are replication incompetent^40^. The carboxyl terminal domain of P21 (150–183), linked to amino acids 141–149, plays a pivotal role in the packaging of pgRNA and subsequent reverse transcription^72–76^. However, the carboxyl terminus, which is shared by P22 but not P20 and P17, is not required for the secretion of empty virions^44^. Thus the formation of heterocapsids composed of P21 and P20 or P17 would lead to the formation of empty virions, which may or may not be enveloped^44^. In addition the P22 that is retrotransported could form alternate weak dimers under the reducing conditions found in the cytosol^66^. Thus these heterocapsids and alternate dimers would be formed at the expense of P21 capsids, decreasing replication leading to the reduced HBsAg expression and secretion observed (Fig. 7). The G1862T mutation causes a retardation of HBeAg expression and has been shown to lead to the accumulation of HBeAg in the ER-Golgi intermediate compartment^39^. This would block the secretory pathway and in turn, decrease the secretion of subviral particles composed of HBsAg, which are also expressed via the ER.

Intracellular trafficking of subgenotype A1 of HBV precore proteins individually was followed using confocal immunofluorescent microscopy. This was unaffected by the CMV promoter and different cell types used for transfection. In the viral context, precore protein expression was affected by absence of core, and affected HBsAg expression, suggesting an interrelationship between precore proteins, HBcAg and HBsAg. This modulatory role of HBeAg and its precursors may be important in viral persistence and ultimate development of HCC.

## Materials and methods

### Plasmid constructs

Using the subgenotype A1 HBV replication competent clone (pHBV_A1, accession number KM519453)^37^ as the template, the DNA corresponding to HBeAg (p17) or its precursors, P25, P22 and P20 was amplified using primers shown in Supplementary Table S1. The preC/C region was also amplified from the subgenotype A1 HBV replication competent clone with the G1862T mutation (GenBank KM519452.1). The polymerase chain reaction (PCR) products were cloned into the pcDNA3.1 vector (Invitrogen, by Thermo Fisher Scientific, Waltham, Massachusetts, USA, pcDNA™3.1( +)). Plasmids pcDNA_A1P25, pcDNA_A1P22, pcDNA_A1P20 and pcDNA_A1P17 coded for proteins p25, p22, p20 and p17, respectively. Plasmid pcDNA_A1P25m encoded p25 (G1862T), pcDNA_A1P22*, pcDNA_A1P20* and pcDNA_A1P17* were generated by site-directed mutagenesis of the second start codon of preC/C ORF (1901–1903: ATG to ACG, change from Met to Ile) on each of plasmids pcDNA_A1P25, pcDNA_A1P22, pcDNA_A1P20 and pcDNA_A1P17. This mutation allows the expression of HBeAg and its precursors, without expression of core protein.

pcDNA_BCP_p25: p25 expressing plasmid driven by authentic HBV promoter. The BCP region and the region encoding p25 was restricted from the HBV overlength clone, pHBV_A1 using *XbaI*/*NcoI* (New England Biolabs), which cut before the BCP region and after the P25 gene. The digested product was loaded on a 1% agarose gel and gel purified with Macherey–Nagel Nucleic Acid and proteins purification kit.

pHA_A1P22 was obtained by cloning P22 gene in pKH3 plasmid, between *BamHI* and *KpnI* sites, after the HA tag, to express the HA tag in Nterminal of P22. pA1P22_HA was obtained by cloning P22 gene in pKH3 plasmid, between *Hind*III and *SalI* sites, before the HA tag, to express the HA tag in C-terminal of P22.

Plasmids pHBV_A1preC- and pHBV_A1C- were generated by site-directed mutagenesis of the first (1814–1816) and second (1901–1903) start codons of preC/C ORF on the plasmid pHBV_A1, respectively. pHBV_A1preC- allows the expression of all the HBV proteins except HBeAg and its precursors because of the mutation of the first start codon of preC/C ORF (ATG to ACG, change from Met to Ile).pHBV_A1C- allows the expression of all the HBV proteins except core protein because of the mutation of the start codon of C ORF (ATG to CTG, converting Met to Leu).

psfGFP-Golgi-7 (Addgene, Watertown, Massachusetts, USA, 56,485) contains the Golgi located protein 1,4-galactosyltransferase fused to eGFP, under the control of a CMV promoter.

Plasmid pcep21 contains the core gene genotype D expressed under control of the CMV promoter (kind donation of Prof. Kann, University of Gothenburg).

### Cell line and DNA transfections

Protocols followed were as described by Deroubaix et al.^68,77^. HuH-7 cells were maintained in Dulbecco’s Modified Eagle’s medium (DMEM, Gibco, by Thermo Fisher Scientific, Waltham, Massachusetts, USA) supplemented with 10% (v/v) fetal calf serum, penicillin (100U.mL^−1^) /streptomycin (100 µg.mL^−1^) (complete medium).

For immunofluorescence analysis, 180,000 HuH-7 or HepG2 cells were seeded on coverslips, in 12-well dishes, in complete medium and incubated for 24 h at 37 °C, 5% CO_2_ in air. Then, cells were transiently transfected with each plasmid (1 μg DNA in serum-free DMEM), using 3 µl reagent (TransIT-LT1 Transfection Reagent, Mirus Bio Corporation, Madison, Wisconsin, USA) following the manufacturer’s protocol. Transfection was done in complete medium and cells were incubated at 37 °C, 5% CO_2_ in air. Further analyses were carried out at 6, 12, 18 h, 1 day, 3 days and 5 days post-transfection.

For western blot analysis, 3.7 million HuH-7 cells were seeded in 10 cm dishes. 24 h later, they were transfected with each of the plasmids (20.7 µg DNA) following the TransIT-LT1 manufacturer’s protocol and harvested at days 1, 3 and 5 post-transfection. Non-transfected cells constituted the negative control, and cells transfected with an eGFP expressing plasmid constituted the positive control. Pellets were lysed with RIPA buffer and sonicated (3 × 30 s on ice). Extracts were analysed by western blot.

For enzyme linked immunosorbent assay (ELISA) analyses, transfections were performed in 12 well plates (as for immunofluorescence). 180 000 cells were seeded in each well of a 12 well plate. 24 h later, 1 µg DNA was transfected following the manufacturer’s protocol. At different times after transfection (1 day, 3 days and 5 days), the supernatant was collected, treated with protease inhibitors (Roche Diagnostics GmbH by Roche Applied Science, Mannheim, Germany, cOmplete, Mini, EDTA-free Protease Inhibitor Cocktail Tablets) and stored at − 20 °C. The cells were harvested from each well after trypsinization (Gibco by Thermo Fisher Scientific, Waltham, Massachusetts, USA, Trypsin–EDTA (0.25%), Phenol Red), washed in cold phosphate buffered saline (PBS 1X) (Gibco, by Thermo Fisher Scientific, Waltham, Massachusetts, USA, PBS tablets) and pelleted following centrifugation at 800 rpm. To lyse the cell pellets, 200 μl of RIPA buffer containing protease inhibitors was added for 5 min. The cells were sonicated 5 times (10 s ON/30 s OFF) and centrifuged at 12 000 rpm. Supernatants were collected and stored at − 20 °C or used immediately for ELISA.

### Antibodies

For immunofluorescence, the rabbit polyclonal antibody raised against core protein (DAKO^57^, Agilent Technologies, Santa Clara, California, USA, Polyclonal Rabbit Anti-Hepatitis B Virus Core Antigen, 1/1000) or anti-HA tag antibody (Merk Millipore, Kenilworth, New Jersey, USA, 1/800) were used, followed by AlexaFluor 488 goat anti-rabbit, (Molecular Probes, by Thermo Fisher Scientific, Waltham, Massachusetts, USA, 1:1000). For western blot, DAKO anti-core antibody and Horseradish Peroxidase-labelled secondary antibody were used.

### Western blots

The protocol was used as described previously^38^, with some modifications. 15 µg of protein extracts were separated on 12% or 15% SDS–polyacrylamide gels and blotted onto nitrocellulose using a wet transfer (Bio-Rad Laboratories, Hercules, California, USA), following the manufacturer's instructions. Blocking was done 1 h at room temperature or overnight at 4 °C with tris-buffered saline (TBS), 0,1% Tween-5% milk. HBeAg and precursors were detected with DAKO anti-core antibody (Agilent Technologies, Santa Clara, California, USA) 1/10 000 for 2 h at room temperature. The membrane was washed 3 times with TBS-Tween and incubated 1 h at room temperature with anti-rabbit secondary antibody linked to horse radish peroxidase (HRP) (1/10 000, Bio-Rad Laboratories, Hercules, California, USA). The membrane was washed 3 times with TBS-Tween and incubated 1 min with SuperSignal West Femto Maximum Sensitivity Substrate (Thermo Fisher Scientific, Waltham, Massachusetts, USA).

#### Equipment and settings

The western blots were viewed with the Gel Doc XR (Bio-Rad Laboratories, Hercules, California, USA). Iris, focus and zoom were adjusted to have a clear picture. Image of the blot was acquired with the Quantity One software after two exposure times (chemiluminescence mode, one minute, exposure 1 and 30 s, exposure 2). The white light Epi-illumination from the white light transilluminator was used to capture the image of the molecular weight marker. The picture from chemiluminescence and the one from the white light Epi-illumination were then overlapped to determine the size of the bands.

### ELISA

Expression of HBeAg and its precursors in cells (lysates) and their secretion in the culture media (supernatants) were analysed with an ELISA kit (ELISA kit: ETI-EBK PLUS (HBeAg), (DiaSorin, Saluggia, Italy) according to the manufacturer’s protocol. The concentrations were determined in OD/µl for the transfection of each construct expressing HBeAg and/or its precursors.

### Immunofluorescence

Protocols followed were as described by Deroubaix et al.^68,77^. Cells cultured on coverslips were washed 3 times with PBS and fixed with 3.7% formaldehyde in 1X PBS for 10 min at room temperature (RT). The fixed cells were washed 3 times again with 1X PBS and permeabilized with 0.1% Triton-PBS for 8 min and washed 3 times with PBS. Cells were incubated 1 h, at room temperature with 1% bovine serum albumin (BSA, Fraction V, Roche Diagnostics GmbH by Roche Applied Science, Manneheim, Germany) (diluted in PBS 1X). Next, the cells were incubated with the primary antibody for 1 h at 37 °C. Then the cells were washed 5 times with PBS 1X and incubated with the secondary antibody for 1 h at 37 °C. Cells were washed 5 times with PBS 1X. DNA was stained with 4′,6-Diamidino-2-Phenylindole, Dihydrochloride (DAPI, 1 mg/mL, 1/1000, Sigma-Aldrich, Kenilworth, New Jersey, USA) for 10 min at room temperature in the dark. Coverslips were mounted using ProLong Gold Antifade Mountant (Invitrogen, by Thermo Fisher Scientific, Waltham, Massachusetts, USA) and analysed by confocal microscopy the following day.

### Microscopy and image analysis

The equipment and settings were as described previously^71^. Microscopy was performed using a Zeiss Laser Scanning Confocal Microscope 780 (Carl Zeiss AG, Oberkochen, Germany), equipped with a 63 X objective (oil immersion alpha Plan-Apochromat 63X/1.40 Oil CorrM27 (Zeiss)) and Zen Blue software 2.1.

#### Equipment and settings

Images were taken in 12 bits, with a sequential, bidirectional acquisition, averaged on 3 images, with an image size of 1024*1024 (pixel size = 0.02 μm). Gain, laser intensity (2%) (488 nm laser for AlexaFluor488 and 405 nm laser for DAPI), with a 1 AU pinhole, were kept constant to obtain comparable results between different slides of an experiment.

High resolution images: Images were taken with the Zeiss Airyscan, in a super-resolution mode, with a 63X objective (oil immersion alpha Plan-Apochromat 63X/1.40 Oil CorrM27 (Zeiss)) and Zen Blue 2.1 software. Images were taken in 12 bits, with a sequential, bidirectional acquisition, averaged on 3 images. The images were then processed to give the high resolution image. The Zen software processes each of the 32 Airy detector channels separately by performing filtering, deconvolution and pixel reassignment to obtain images with enhanced spatial resolution and improved signal to noise ratio. Z-stacks and 3D reconstruction were performed using Zen Black 2.1 software.

Quantification of fluorescence was performed using ImageJ software (Fiji/Image J, https://imagej.net/Fiji/Downloads), as described by Deroubaix et al.^77^. Briefly, the mean of fluorescence in the nucleus and in the cytoplasm of each transfected cell was determined. This measures the relative protein concentration. The background signal was subtracted for each nucleus and cytoplasm of each transfected cell analysed. The background was determined for each image by calculating the average of the mean of fluorescence of nuclei and cytoplasm of all non-transfected cells. The cells were classified as a function of their ratio of mean of fluorescence between the nucleus and the cytoplasm. If the ratio of nucleus/cytoplasm is below 1.0, this indicates an accumulation of fluorescence in the cytoplasm. If the ratio is above 1.0, there is an accumulation of fluorescence in the nucleus. The cells were then classified in a graph showing the percentage of cells having an accumulation in the cytoplasm, in the nucleus or having an equal distribution between nucleus and cytoplasm for n cells.

### Statistical analysis

Statistics were realized by using a two sample t-test (pooled variance) (GraphPad Prism 9.0.0, https://www.graphpad.com/quickcalcs/ttest1.cfm), to verify if the average of the means of the ratios of fluorescence between the nucleus and the cytoplasm (R = N/C) are the same or different between two time-points of the kinetic (Fig. 3) for n = 3 or n = 4 experiments. T-test was also done to check if the mean of HBeAg secretion is the same between the transfection with pHBV_A1 and pHBV_A1C- for ELISA test (Fig. 6). The tests were two-tailed, n = 3, and the significance level α = 0.05.

## Supplementary Information

Supplementary Information

## Data Availability

The datasets generated during and/or analysed during the current study are available from the corresponding authors on reasonable request.
