# Supplementary material for: In vitro expression of precore proteins of hepatitis B virus subgenotype A1 is affected by HBcAg, and can affect HBsAg secretion
Source: Sci Rep. 2021 Apr 14;11:8167. doi: 10.1038/s41598-021-87529-9 (PMC8046783; doi:10.1038/s41598-021-87529-9)
Supplement: Supplementary file 1 — Supplementary Information [file 41598_2021_87529_MOESM1_ESM.pdf]

Supplementary information

In vitro expression of precore proteins of hepatitis B virus subgenotype A1 is affected by HBcAg, and can affect HBsAg secretion

Aurélie Deroubaix[1,2 *] and Anna Kramvis[1*]

Hepatitis Virus Diversity Research Unit, Department of Internal Medicine, School of Clinical Medicine[1] and Life Sciences Imaging Facility[2], Faculty of Health Sciences, University of the Witwatersrand, Johannesburg, South Africa.

Target	Sequences	Template	Final product
P25, P25m PCR products	5' CTA TGC GGC CGC GCC ACC ATG CAA CTT TTT CAC CTC T 3'<br>5' CCA CGGATC CGC CAT GGC CTA ACA TTG AGA TTC CCG AG 3'	pHBV_A1	pcDNA_A1P25, pcDNA_A1P25m
P22 PCR product	5' CTA TGC GGC CGC GCC ACC ATG TCC AAG CTG TGC CTT G 3'<br>5' CCA CGGATC CGC CAT GGC CTA ACA TTG AGA TTC CCG AG 3'	pHBV_A1	pcDNA_A1P22
P20 PCR product	5' CTA TGC GGC CGC GCC ACC ATG TCC AAG CTG TGC CTT G 3'<br>5' CCA CGG ATC CGC CAT GGC CTA TCT TCG TCT GCG AGG CGA 3'	pHBV_A1	pcDNA_A1P20
P17 PCR product	5' CTA TGC GGC CGC GCC ACC ATG TCC AAG CTG TGC CTT G 3'<br>5' GAA GGG ATC CGC CAT GGC CTA TCT AGG GGA CCT GCC TCG GT 3'	pHBV_A1	pcDNA_A1P17
Site-directed mutagenesis ATG precore	5' CTG CGC ACC ATC ATC CTG CAA CTT TTT CAC 3'<br>5' GTG AAA AAG TTG CAG GAT GAT GGT GCG CAG 3'	pHBV_A1	pHBV_A1preC-
Site-directed mutagenesis ATG core	5' GAT GGC TTT GGG GCC TGG ACA TTG ACC CT 3'<br>5' AGG GTC AAT GTC CAG GCC CCA AAG CCA TC 3'	pcDNA_A1P25, pcDNA_A1P25m, pcDNA_A1P22, pcDNA_A1P20, pcDNA_A1P17, pHBV_A1	pcDNA_A1P25*, pcDNA_A1P25m*, pcDNA_A1P22*, pcDNA_A1P20*, pcDNA_A1P17*, pHBV_A1C-

Supplementary Table S1: List of primers for PCR and site-directed mutagenesis.

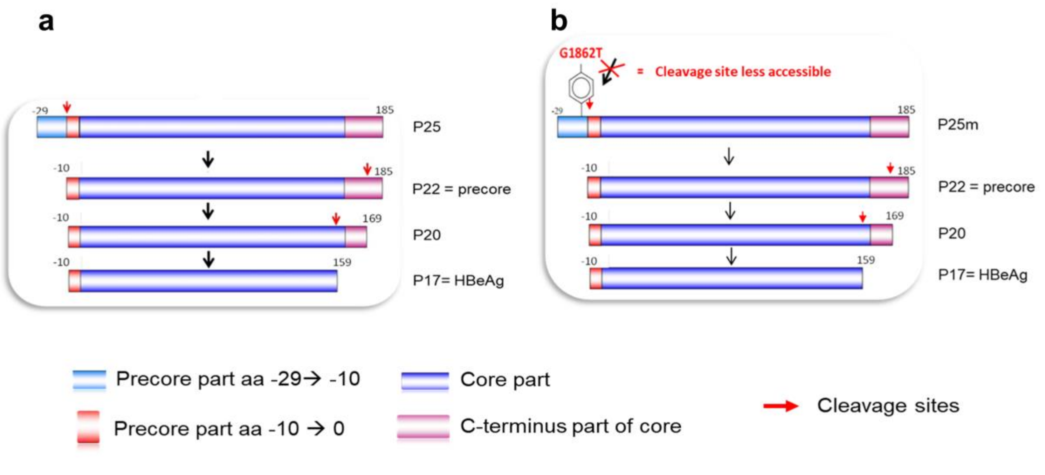

Supplementary Figure S1: Expression of HBeAg and precursors. Schematic representation of post-translational modifications of P25, leading to HBeAg. a. Transfection with **pcDNA_A1P25**. b. Transfection with **pcDNA_A1P25m**.

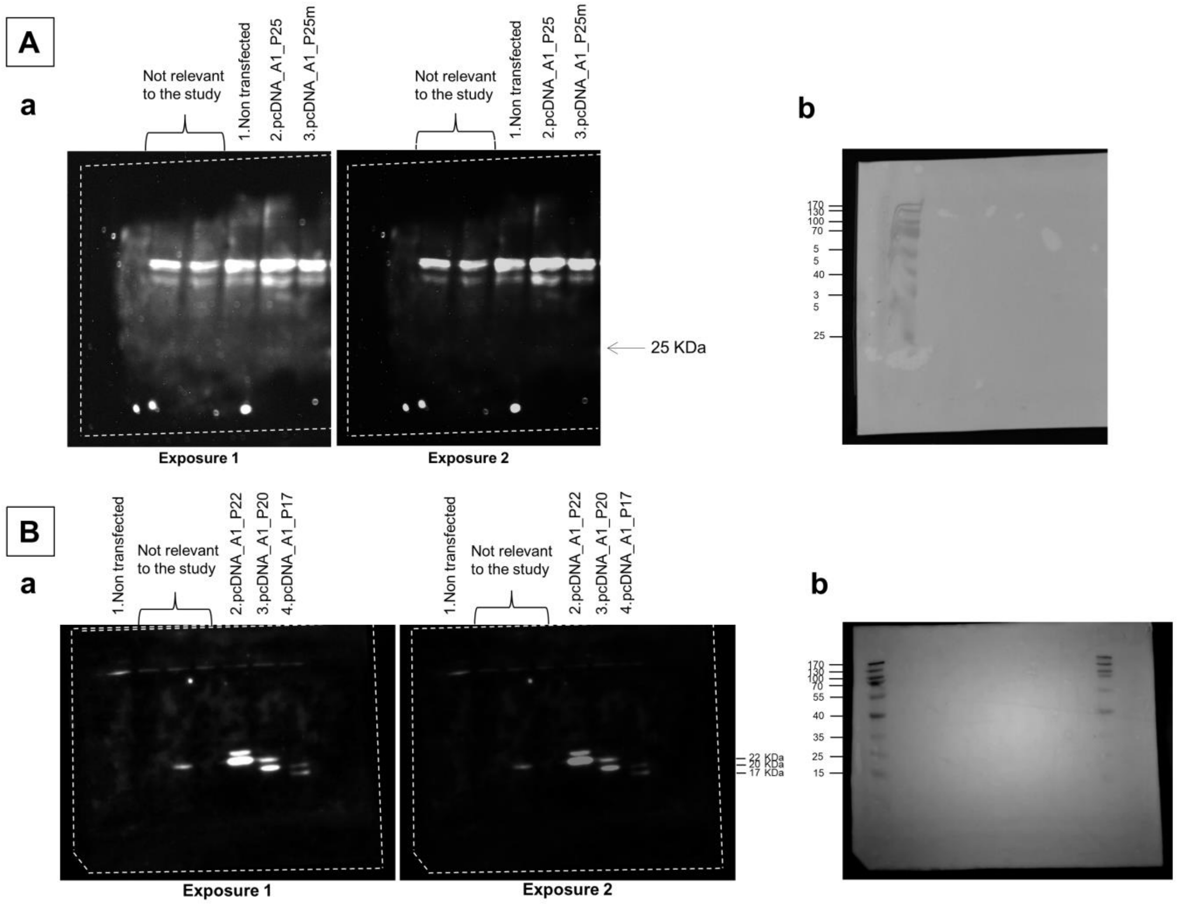

Supplementary Figure S2: Expression of HBeAg and precursors (full length gels). A(a) (full length gel for Figure 1a) and B(a) (full length gel for Figure 1b), chemiluminescent results with the Bio-Rad Gel Doc XR, at two different time exposures (exposure 1= 1 minute, exposure 2= 30 seconds). The shorter exposure time allows decreasing the background. Images with the highest exposure time (Exposure 1) have been cropped and used for Figure 1. One minute exposure time was necessary to observe P25 and P25m as they are expressed in very low quantities. Technically, HuH-7 cells were transfected with the plasmids indicated at the top of the figures. “Non transfected”= non transfected cells, are the negative control. The positive control was not possible for this study as there is no control plasmid for the expression of HBeAg and precursors. Cell lysates were analysed with 15% acrylamide gels. The blots for P25/P25m (A) and P22/P20/P17 (B) were done separately, but did not affect the analysis as we did not perform any quantitative analysis. Western blots were performed with anti-core antibody (DAKO) and a secondary antibody linked to horse radish peroxidase

(HRP). Blots were visualized with Bio-Rad Gel Doc system and pictures were acquired with Quantity One Software. White dashed lines indicate delineations of the membrane. A(b): same membrane as A(a) but shows the molecular weight marker, visualized with white light Epi-illumination. B(b): same membrane as B(a) but shows the molecular weight marker with Epi-illumination. A(a) and A(b), as well B(a) and B(b) were superimposed to determine the size of the bands. “Not relevant to the study”: wells present in the gel but which have to be disregarded because they are not part of the study.

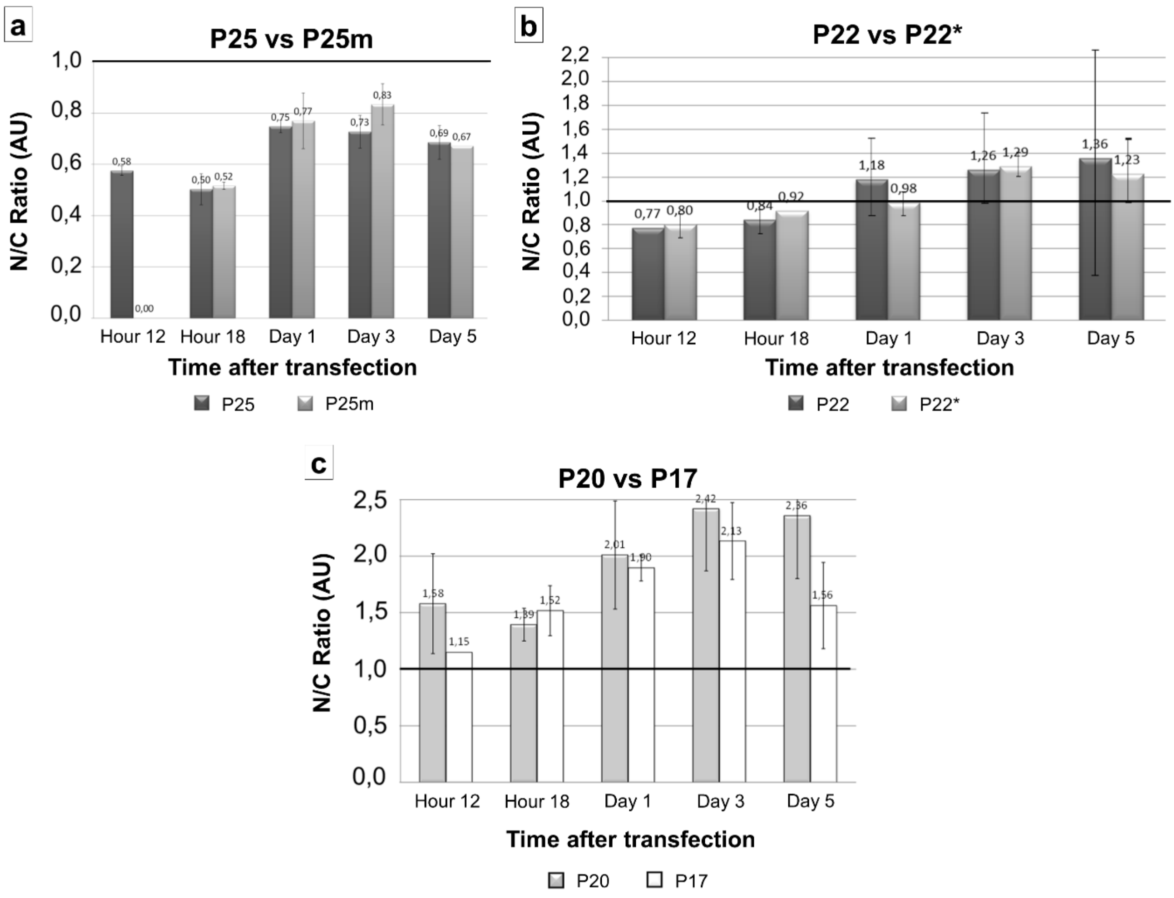

Supplementary Figure S3: Ratios of fluorescence Nucleus/Cytoplasm (N/C) of HBeAg and precursors over time. The ratios of mean of fluorescence Nucleus/Cytoplasm for the cells transfected with HBeAg and precursors from figure 2B were averaged. (a) Comparison of P25 and P25m. (b) Analysis of P22 and P22* (c) Analysis of P20 and P17. Black line: limit where N/C=1 (equal distribution between nucleus and cytoplasm, above: nuclear accumulation, below: cytoplasmic accumulation).

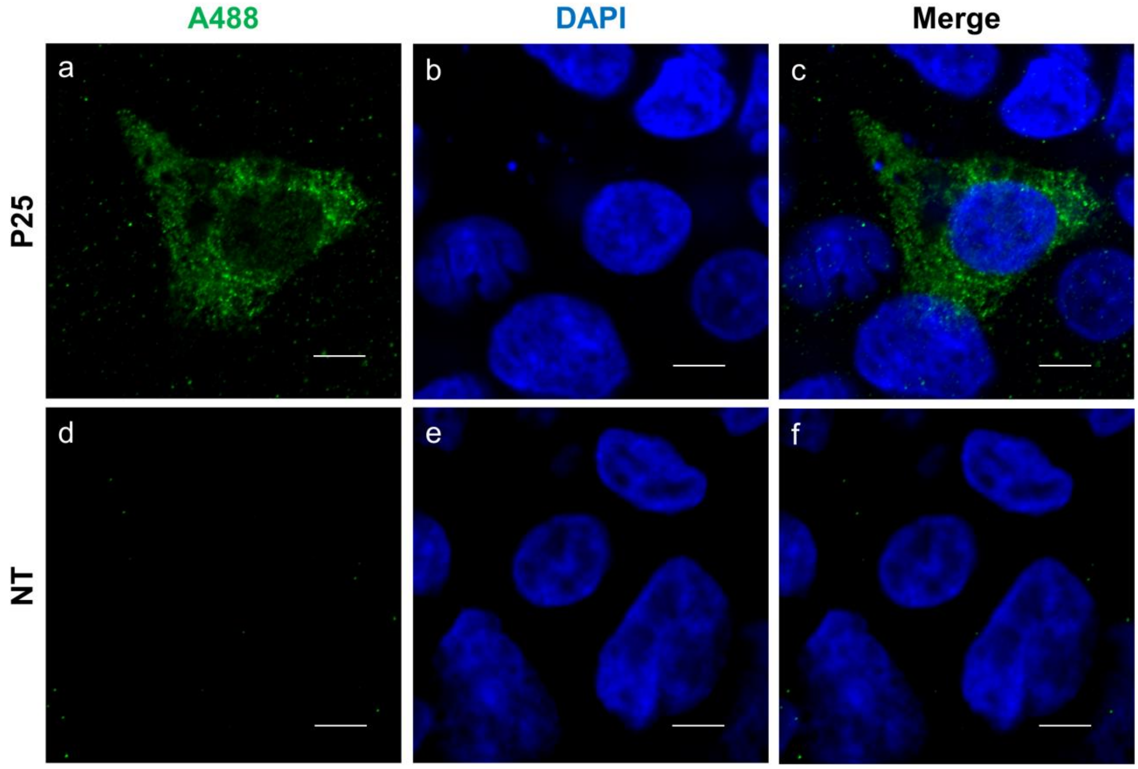

Supplementary Figure S4. Expression of HBeAg and precursors with natural promoter. HBV A1 genome has been digested by *Xba* and *Nco*I in order to have the DNA sequence corresponding to HBV P25 and the PreC/C promoter. The DNA fragment was purified (pcDNA_BCP_p25) and transfected into HuH-7 cells (a-c). Cells were fixed at day 3 post transfection and stained with DAKO anti-core antibody and anti-rabbit antibody conjugated to Alexa 488. Transfected cells were viewed under the confocal microscope. Blue staining: DAPI (b,e); merge pictures: c,f. Bars: 10 µm.

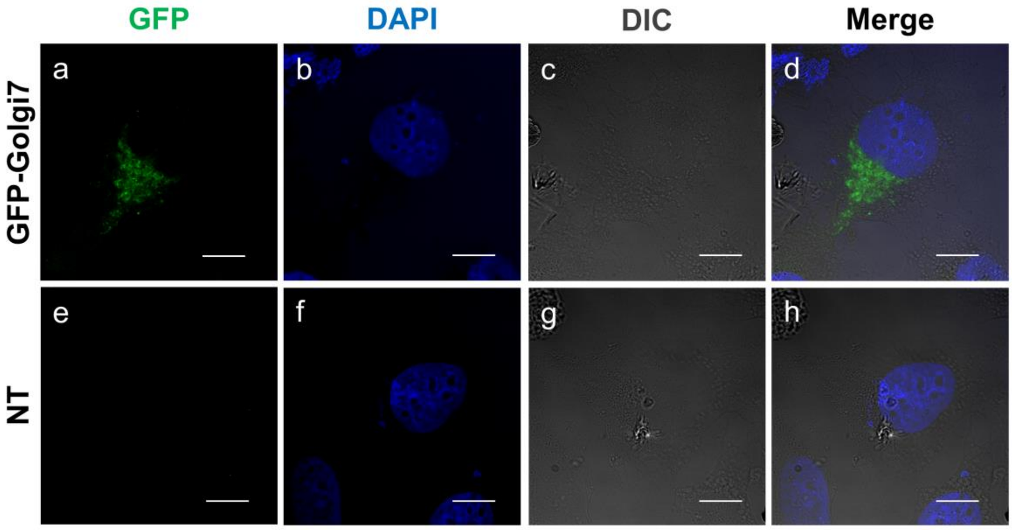

Supplementary Figure S5. Over-expression of Golgi7 protein does not change its localization. HuH-7 cells were transfected with pGFP-Golgi7 (eGFP-1,4-galactosyltransferase under the CMV promoter) (a-d), fixed 3 days post transfection, and stained with DAPI (b,f). Merge: d,h. Differential Interference Contrast (DIC): c,g. NT: non transfected cells (e-h). Bars: 10 µm.

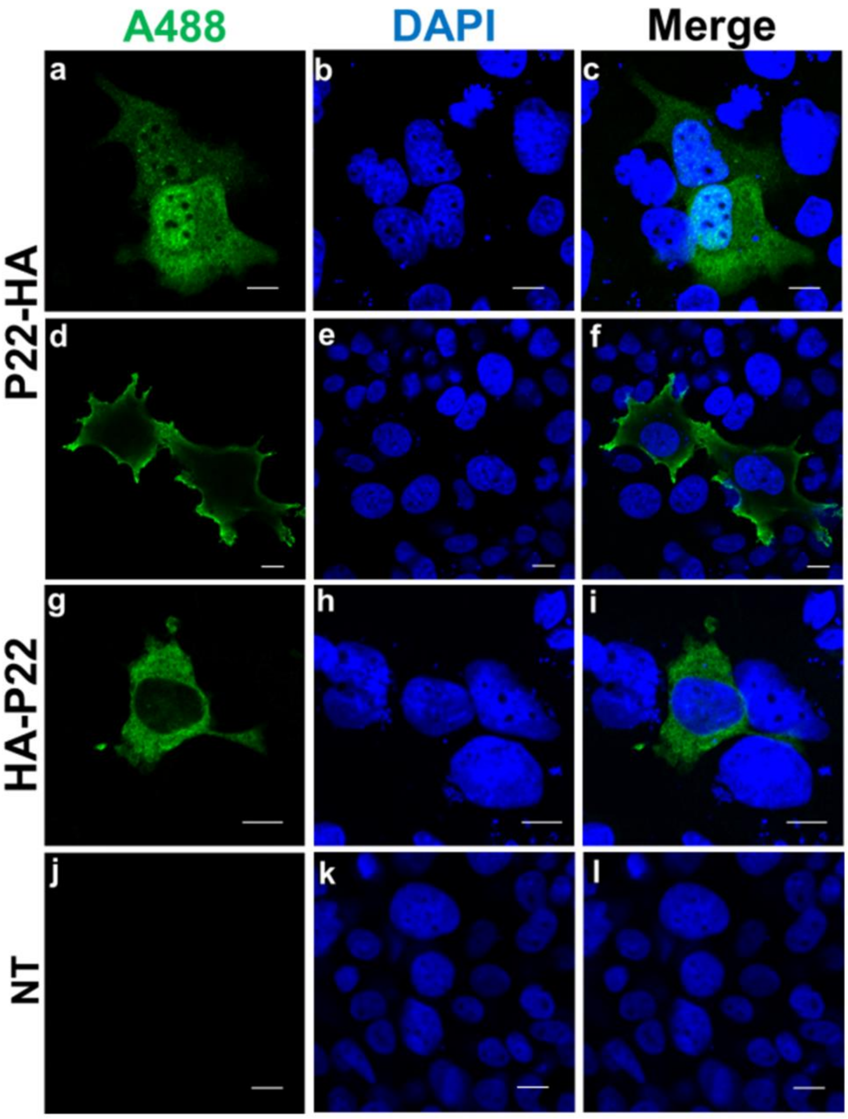

Supplementary Figure S6: Intracellular localization of HA_P22 and P22_HA. After transfection of HuH-7 with **pA1P22_HA** (a-f) and **pHA_A1P22** (g-i), cells were immunostained with an anti-HA antibody (a,d,g,j) and viewed at the confocal microscope at day 3 post-transfection. Nuclei were visualized by DAPI staining (b,e,h,k) ; merge (c,f,i,l). NT: non-transfected cells.

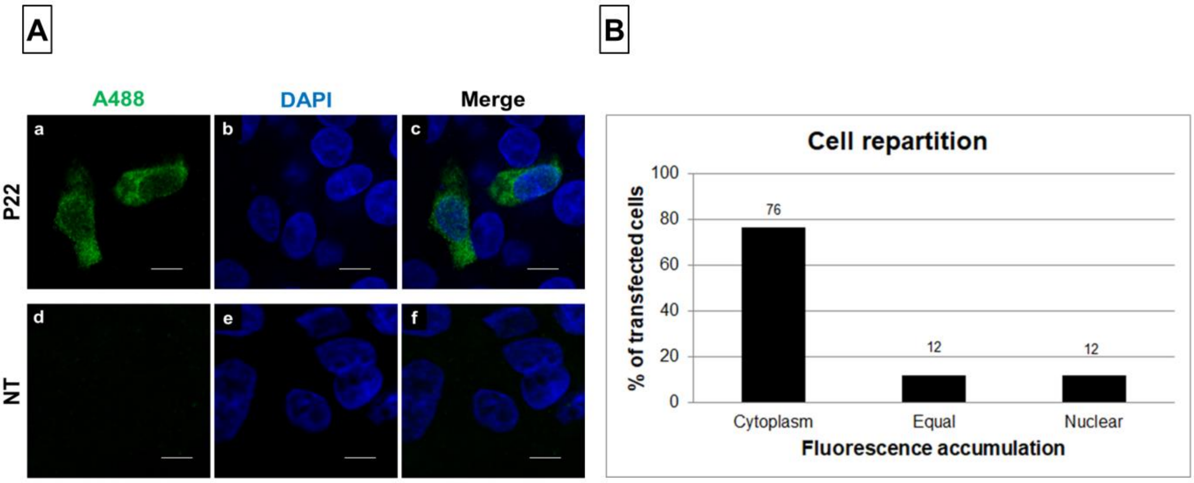

Supplementary Figure S7. Localization of HBeAg and its precursors in HepG2 cells A. pcDNA_A1P22* plasmid, expressing HBV P22 (with lack of core protein expression), was transfected in HepG2 cells. Cells were immunostained with a polyclonal rabbit anti-HBc antibody (DAKO, (a,d)) and viewed with a confocal microscope at 12h post-transfection. Nuclei were visualized by DAPI staining (b,e) ; merge (c,f). B. Quantification of the fluorescence in the nucleus and cytoplasm of transfected cells (n=20) with image J and classification of the cells depending on their Nucleus/Cytoplasm (N/C) ratios. If N/C>1, the proteins localize preferentially in the nucleus; if N/C<1, the proteins localize preferentially in the cytoplasm; if N/C=1, the proteins have an equal distribution between the nucleus and the cytoplasm. Bars: 10 µm.
